# Outpatient Palliative Care Service Involvement: A Five-Year Experience from a Tertiary Hospital in Switzerland

**DOI:** 10.1089/pmr.2023.0052

**Published:** 2024-01-05

**Authors:** Dorothea Rebekka Birkner, Markus Schettle, Markus Feuz, David Blum, Caroline Hertler

**Affiliations:** ^1^ University of Zurich, Zurich, Switzerland.; ^2^ Department of Radiation Oncology, Competence Center Palliative Care, University Hospital Zurich, Zurich, Switzerland.

**Keywords:** caregiver, early implementation, outpatient needs, palliative care, quality of life

## Abstract

**Background:**

The value of early integration of palliative care has been demonstrated increasingly for the past years in both oncological and nononcological diseases. Outpatient palliative care services might represent a feasible approach to implement supportive care in early disease. In this study, we aimed at evaluating which patients use and benefit from outpatient palliative care services, which symptoms are addressed most, and which support services are installed in this early phase of disease.

**Methods:**

We retrospectively analyzed the entire patient collective of a recently developed palliative care outpatient clinic within the leading university hospital in Switzerland for a period of five years. Sociodemographics, symptoms, and information on disease as well as patient-reported outcomes were retrieved from the electronic patient files. Demographic and clinical data were analyzed by descriptive statistics between groups and survival was analyzed by means of Kaplan–Meier estimates and log-rank test.

**Results:**

We report on 642 consultations of 363 patients between 2016 and 2020. Patients had a mean of 1.8 visits (range 1–10), with *n* = 340 patients (93.7%) of patients suffering from an oncological disease. Overall symptom load was high, with *n* = 401 (73.7%) of patient-reported outcomes reporting two or more symptoms. Distress levels of 5 or higher were reported in *n* = 78 (30.4%) of available patient-reported outcomes. Independent of the origin of primary disease and the length of the disease trajectory, patients were referred to the palliative care service in median only four months before death.

**Conclusion:**

We identify high symptom load and distress in the outpatient palliative patient population. Patients benefitted from supportive medication, improvement of ambulatory support systems and advance care planning, and more than one-third of patients remained in follow-up, indicating a good acceptance of the service. Overcoming the overall late referral could, however, further increase the quality of life at earlier stages of disease.

## Background

Owing to incorrect synonymization of palliative and (inpatient) hospice care, the early needs of palliative care patients often remained unaddressed, eventually leading to care gaps during critical periods of worsening illness and deterioration.^1,2^ Within the past years, the value of early integration of palliative care has been acknowledged, underlining the need to focus on the patients' and relatives' quality of life, and also demonstrating a positive impact on patient outcomes as well as an overall lowering of health care costs.^3–5^ Within this modernized conceptualization of palliative care, the interest in services providing early comprehensive palliative care support has increased.^6,7^ A recently published overview of different nonhospice palliative care interventions confirmed the benefit of early and nonhospice disease management.^1^

In this context, an outpatient setting has been identified as an ideal opportunity for early collaborative palliative care support and improvement of continuity and coordination of care.^8–10^ Outpatient clinics represent a key point of entry for timely and continuously access to palliative care already at early stages of palliative diseases.^11,12^ Ideally, outpatient consultations in palliative care include assessing physical and psychosocial symptoms, establishing goals of care, assisting with decision making regarding treatment and coordinating care based on the individual needs of the patient, at an early stage of disease.

The consultation should involve different professions to cater to all needs, and request further assistance at low threshold. The advantage of the outpatient setting is that it requires relatively few resources to address these diverse issues, and that it may provide important and helpful support benefitting patients. Eventually, those support systems will outlast throughout the disease trajectory and relieve burden from the patient and caregiver in the later stages of disease. In addition, they can relieve the primary physician.

However, outpatient palliative care clinics have emerged only in recent past, and only few characterizations of such clinics have been published, especially in Switzerland.^13^ Hence, broad experience about the patients' needs and opportunities to offer support in this specific setting is lacking.^14,15^

In this study, we report on the experiences of the interprofessional outpatient clinic at the University Hospital of Zurich (USZ) in a retrospective analysis of five years. We characterize the patients and caregivers who were mainly referred, and address the questions of specific needs and burden of patients in this early outpatient setting, with the overall aim to identify patients with need for longitudinal support. Eventually, we attempt to a framework of ideal timing for early palliative care implementation, and possible recommendations for referring physicians.

## Materials and Methods

### Data source

**FIG. 1.**
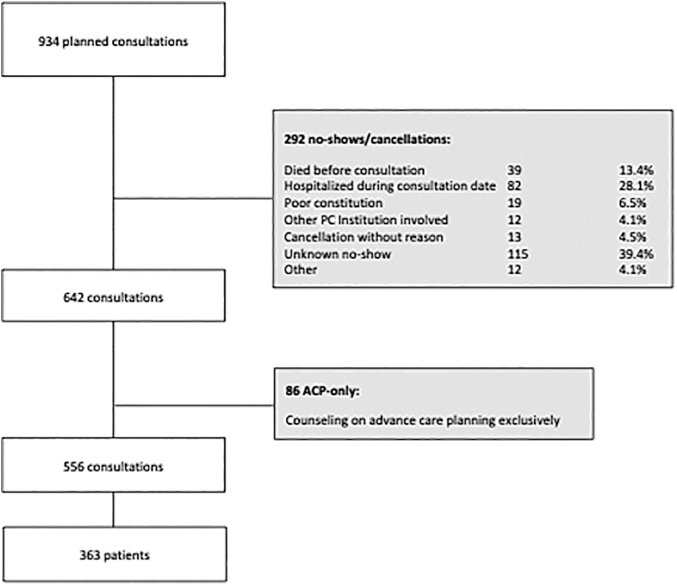
Consort diagram. Total numbers of consultations and patients identified and included or excluded in analyses. ACP, advance care planning; PC, palliative care.

We conducted a retrospective single-center cohort study of patients >18 years that presented at the outpatient clinic of the Competence Center Palliative Care at the USZ, Switzerland, between 2016 and 2020, with the primary goal to characterize which patients are referred to us, and to define the main reasons for referral. Furthermore, we analyzed which support systems were provided in the consultation and aimed to determine differences in symptoms and needs based on the primary diagnosis. We identified 934 planned consultations (Fig. 1). Electronic patient files were the primary source of data acquisition.

### Characteristics of the service

The outpatient palliative care service at the USZ was established in 2015 to ensure a continuity of care to known palliative care patients and to enable an early integration of palliative care for patients from other departments, by facilitating palliative care referrals beyond hospitalization. It is part of the USZ, to date the first-ranked University Hospital in the country, comprising 900 beds, with 42,000 inpatient and >600,000 outpatient contacts per year. Referral occurs from other departments of the hospital, by other hospitals, or by general practitioners, but also by patients and caregivers themselves.

The structure and goals of each consultation differs based on the needs and wishes of the patient and caregiver, in a person-centered approach. In general, addressed topics include assessing physical and psychosocial symptoms, establishing goals of care, assisting with decision making regarding treatment and coordinating care based on the individual needs of the patient.

The interprofessional consultation is held by a specialized palliative care nurse and a specialist palliative care physician. If indicated, further disciplines can be actively involved within the visit (eg, nutritional therapy, psychology, and spiritual care), usually in follow-up visits. Clinic hours are scheduled on two half-days a week with a duration of one hour each, for patients alone or accompanied by their relatives; rarely consultations took place with relatives only, if the patient was too weak to present but agreed to sending a relative. In selected cases, video calls were installed if requested by the patient.

### Patient characteristics and symptom assessment

We obtained data on patient demographics, number of visits, referral clinic, primary disease, and clinical characteristics, including distress level, symptom load, and specific symptoms. In addition, we recorded advance directive, information on the patient network, relatives, and established medication and changes in symptom management medication before and after the consultation. Symptom load was assessed qualitatively and quantitatively according to the number of reported symptoms. Edmonton Symptom Assessment System (ESAS) was used for quantitative patient-reported outcome.

It is a validated multi-item patient-reported outcome measure covering physical and psychosocial symptoms, allowing for mentioning of other symptoms, which is completed either by the patient or by proxy, and sent to the patient beforehand to allow for a comprehensive overview.^16^ It is scored on a 10-point Likert scale. Any indication of a symptom leads to addressing of the burden. Any report ≥5 is considered a high burden. The SENS Model (German acronym for symptoms, decision making, network, and support) was used to assess symptoms and needs on a structured qualitative level.^17^

It is a concept allowing for a semistructured assessment of topics relevant in palliative care, encompassing definitions of the WHO for palliative care and National Comprehensive Cancer network (NCCN) foci. Distress level was assessed by means of distress thermometer, a patient-reported tool using a 0 to 10 rating scale developed by the NCCN. The same data were obtained also for the patients who refused a visit, to allow for a potential comparison of the populations. For analysis, an encounter-level approach instead of a patient-level approach was used to avoid false multiplication of symptom reports. Finally, we documented date of initial diagnosis, date of referral, and dates of death, respectively, date of last contact.

### Statistics

Demographic and clinical data were analyzed by descriptive statistics. We calculated mean and standard deviation for all continuous variables. The chi-square test was performed for analysis of nominal variables, and the Mann–Whitney *U* test was used for the comparison of ordinal variables between groups. Survival was calculated from time of consultation to death; overall survival was calculated from time of diagnosis to death; and survival was analyzed by Kaplan–Meier estimates. Kaplan–Meier curves were compared using the log-rank test. Patients without event (death) were censored at time of last follow-up. For statistical analysis, SPSS Version 28 was used (SPSS IBM Corp., Armonk, NY).

### Ethics

The study was conducted in accordance with the Declaration of Helsinki and applicable regulatory requirements and has been approved by the local ethics committee (KEK-ZH-Nr. 2019-02488).

## Results

### Patient-related aspects

Between January 2016 and December 2020, a total of 363 patients were seen in 642 consultations. Mean number of consultations was 1.8, with a range of 1 to 10 consultations per patient. The mean age was 65 years, and 155 (42.7%) were females. Most of the patients were of Swiss nationality (79.3%), with a general insurance (58.6%) and suffered from an oncological primary disease (94.2%). Further patient characteristics are summarized in Table 1 and Supplementary Table S1.

**Table 1.** Group Comparison by Main Diagnosis

	Oncological diagnosis (***N*** = 340), ***N*** (%)	Nononcological diagnosis (***N*** = 23), ***N*** (%)	***p***
Age (years)			
Median	66.0	67.0	0.308
Range	19.0–98.0	26.0–86.0	
Age groups			
<50	45 (13.2)	8 (34.8)	
50–69	107 (31.5)	3 (13.0)	**0.018**
70–79	85 (25.0)	7 (30.4)	
>80	103 (30.3)	5 (21.7)	
Sex			
Male	196 (57.6)	12 (52.2)	0.666
Female	144 (42.4)	11 (47.8)	
Main symptom			
Pain	183 (56.0)	9 (39.1)	
Dyspnea	20 (6.1)	5 (21.7)	**0.037**
Fatigue	41 (12.5)	5 (21.7)	
Neurological	40 (12.2)	1 (4.3)	
Psychoemotional	33 (10.1)	3 (13.0)	
None	10 (3.1)	0 (0)	
Other	10 (—)		
Symptom load			
Little	58 (17.8)	2 (11.1)	
Moderate	170 (52.3)	9 (50.0)	0.588
Strong	87 (26.8)	7 (38.9)	
Extreme	10 (3.1)	0 (0)	
Missing	15 (—)	5 (—)	
Living situation			
Alone	98 (28.8)	6 (26.1)	0.779
Supported	242 (71.2)	17 (73.9)	
Advance directives			
Preexistent	160 (54.2)	9 (52.9)	**0.010**
Within consultation	47 (15.9)	7 (41.2)	
None	88 (29.8)	1 (5.9)	
Missing	45 (—)	6 (—)	

Bold numbers indicate *p* < 0.05.

Data on cancellations and no-shows were collected accordingly for 244 of 292 no-show patients. This patient population was characterized by mainly oncological diagnosis as well (92.9%), yet the repartition of the origin of cancer differed from those of the patients who visited (*p* < 0.001). Although basic socioeconomics were similar in both groups, the no-show cohort comprised fewer elderly patients (*p* < 0.001) (Supplementary Table S1). Reasons for cancellation remained often unknown (39.4%); one-third (28.1%) of patients were already hospitalized at the time of the planned consultation, and 13.4% of patients passed away before the appointment (Fig. 1).

When investigating potential sex-related factors, men and women visiting our clinic did not differ significantly regarding symptom load or advance directives. Unsurprisingly, there was a typical repartition difference in some cancer origins by sex (more breast cancer in female and urological cancer in male; *p* < 0.001). However, women were less often supported in their living situation, and one-third of female patients lived alone without assistance (*p* = 0.007) (Supplementary Table S2). Another sex-related disbalance was detected regarding the caregivers of the patients. We confirmed a high number of visits together with a caregiver in 471 (73.4%) visits. Carers were mostly partners (*n* = 310, 65.8%) or children (*n* = 85, 18.0%), and female for the biggest part (*n* = 307, 65.2%; data not shown).

### Disease-related aspects

Most patients presented with an oncological diagnosis (*N* = 340, 94.2%). The oncological patient group comprised less younger patients (<50 years) compared with the nononcological group (*p* = 0.018), and more often presented with pain as lead symptom (*p* = 0.037). Advance directives were less frequent in the oncological cohort, and less often the topic of the consultation (*p* = 0.01) (Table 1). Although no significant differences were noted between the disease groups with regard to symptom load, the overall burden was high in both groups, with >50% of patients experiencing at least moderate symptom load, and more than one-fourth reporting strong symptom load in both patient groups (Table 1).

When sorted by leading diagnosis, the primary reported symptom differed between diagnosis groups, with pain and dyspnea being the most frequently reported symptom in lung cancer patients, whereas fatigue was mostly reported by hemato-oncological patients. Interestingly, although most cancer patients reported psychoemotional symptoms as a leading symptom, none of the leading noncancer diagnosis patients reported this (Table 2). In the 257 consultations with documented distress level, *n* = 78 (30.4%) patients located their distress level at 5 or higher (Table 3).

**Table 2.** Symptom Load by Diagnosis

***p*** < 0.001	Leading symptom				
	Pain (***N*** = 192)	Dyspnea (***N*** = 25)	Fatigue (***N*** = 46)	Neurological (***N*** = 41)	Psychoemotional (***N*** = 36)
Brain cancer	14 (7.3)	2 (8.0)	3 (6.5)	**24 (58.5)**	5 (13.9)
Head and neck cancer	**27 (14.1)**	2 (8.0)	4 (8.7)	2 (4.9)	5 (13.9)
Lung cancer	**36 (18.8)**	**6 (24.0)**	7 (15.2)	4 (9.8)	6 (16.7)
Gyneco-oncology	19 (9.9)	1 (4.0)	3 (6.5)	1 (2.4)	4 (11.1)
Gastrointestinal cancer	**28 (14.6)**	1 (4.0)	7 (15.2)	4 (9.8)	6 (16.7)
Prostate cancer	14 (7.3)	3 (12.0)	2 (4.3)	1 (2.4)	1 (2.8)
Sarcoma	8 (4.2)	2 (8.0)	4 (8.7)	1 (2.4)	2 (5.6)
Dermato-oncology	11 (5.7)	0 (0)	3 (6.5)	2 (4.9)	2 (5.6)
Nephro-uro-oncology	11 (5.7)	1 (4.0)	0 (0)	1 (2.4)	1 (2.8)
Hemato-oncology	8 (4.2)	2 (8.0)	**8 (17.4)**	0 (0)	0 (0)
Other (cancer)	7 (3.6)	1 (4.0)	1 (2.2)	0 (0)	1 (2.8)
Cardiology	2 (1.0)	2 (8.0)	1 (2.2)	0 (0)	**0 (0)**
Pneumology	2 (1.0)	1 (4.0)	0 (0)	0 (0)	**0 (0)**
Neurology	1 (0.5)	1 (4.0)	3 (6.5)	1 (2.4)	**0 (0)**
Other (noncancer)	4 (2.1)	0 (0)	0 (0)	0 (0)	3 (8.3)

Bold numbers indicate highest or lowest numbers; numbers of interest.

**Table 3.** Patient-Reported Outcomes

Distress	***n***	%
0		
1	10	3.4
2	17	6.6
3	20	7.8
4	23	9.8
5	17	6.6
6	48	18.7
7	26	10.1
8	34	13.2
9	39	15.2
10	15	5.8

Symptoms	***n***	%
No symptoms	8	3.2
1 symptom	58	23.1
2 symptoms	73	29.1
>2 symptoms	112	44.6

In addition, symptom burden assessment by means of ESAS indicated that in our patient population, more than half of the patients reported two or more burdening symptoms in the first consultation (Table 3). When assessed for any reported symptom, the most frequently reported symptom in the overall patient population were symptoms of the gastrointestinal tract (*n* = 446, 82%), mostly lack of appetite, nausea, and constipation. More than half of the patients (*n* = 309, 56%) reported suffering from insufficiently controlled pain.

**FIG. 2.**
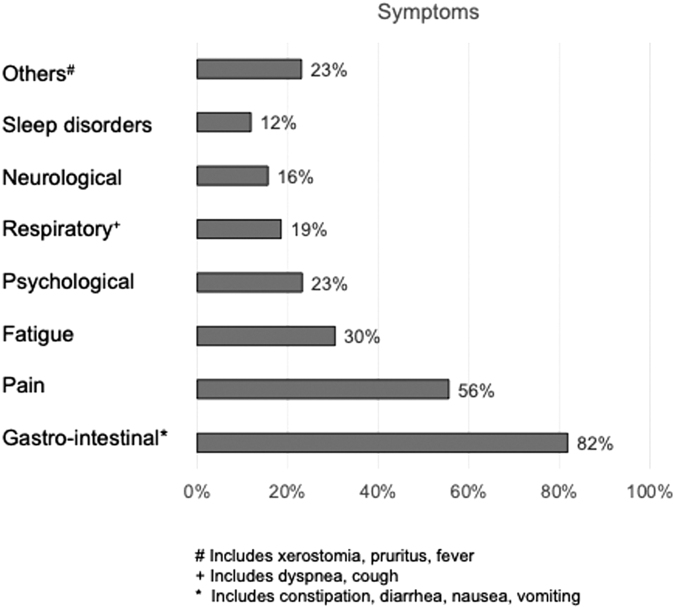
Symptom burden. Overall patient-reported symptoms, percentage of total numbers.

Clustering of all reported symptoms are shown in Figure 2. Interestingly, although overall survival differed significantly between the cohort of oncological and nononcological patients (*p* < 0.001), with a median overall survival of 32 months (confidence interval [CI] 25.5–38.4) in the oncological group versus 129 months (CI 15.8–242.2) in the nononcological group, both patient cohorts presented or were referred to the palliative care outpatient service only months before death in median, irrespective of the primary disease (Table 4 and Supplementary Table S3).

**Table 4.** Survival Data (Log-Rank Analysis)

From consultation				
Survival	***N*** (events)	Median OS (months)	95% CI	***p***
All patients	359 (259)	4.0	3.34–4.66	
Age group				
<50	52 (36)	3.0	0.90–5.10	0.196
50–59	109 (82)	3.0	2.02–4.00	
60–69	91 (75)	3.0	1.97–4.03	
≥70	107 (66)	6.0	3.57–8.43	
Sex				
Male	206 (152)	3.0	2.21–3.79	0.514
Female	153 (107)	4.0	2.79–4.21	
Diagnosis				
Nononcological	23 (15)	7.0	0.00–14.29	0.390
Oncological	336 (244)	3.0	2.37–3.63	
Main symptom				
Pain	189 (140)	3.0	2.10–3.91	0.888
Dyspnea	24 (18)	5.0	0.00–10.31	
Fatigue	46 (32)	3.0	0.82–5.18	
Neurological	41 (31)	3.0	1.70–4.30	
Psychoemotional	36 (24)	3.0	1.49–4.52	
None	10 (7)	7.0	3.04–10.96	
Symptom load				
Little	59 (42)	6.0	3.89–8.11	0.251
Moderate	177 (128)	4.0	3.12–4.88	
Strong	94 (73)	2.0	1.10–2.94	
Extreme	9 (7)	3.0	1.54–4.46	
Living situation				
Alone	103 (73)	4.0	3.25–4.75	0.854
Supported	256 (186)	3.0	2.12–3.88	
Advance directives				
Preexistent	168 (126)	3.0	1.71–4.29	0.618
Within consultation	52 (36)	5.0	2.28–7.72	
None	88 (64)	3.0	1.71–4.229	

CI, confidence interval; OS, overall survival.

### Service-related aspects

Out of the 642 consultations, 363 (56.5%) were first visits and 279 (43.5%) were follow-up visits. In total 86 (13.4%) consultations implied specifically advance care planning (ACP) guidance, 24 (3.7%) were held to counsel relatives in the absence of the patient, and two (0.3%) consultations were held for the relatives after death of the patients, as part of a bereavement dialogue. Documentation of consultations implying ACP setups differed from the standard consultations, focusing on ACP-related topics. Over the years, an increase in the use of the service was noticeable, despite a temporary reduction in consultation numbers in 2020 due to the COVID-19 pandemic.

#### Referral

More than half of the referrals arose from in-house clinicians, mainly from the Departments of Radiation Oncology or Hemato-Oncology (*n* = 166, 45.7%). External referrals were less frequent (*n* = 39, 10.7%), of which 28 (7.7%) were self-referrals from the patients themselves. Referral reasons were heterogeneous and sometimes without a specific question (*n* = 139, 38.3%). Symptom management (*n* = 50, 13.8%), ACP (*n* = 60, 16.5%), and counseling about best supportive care (*n* = 70, 19.3%) were frequently documented referral reason.

#### Advance care planning

A total of 86 consultations were related to ACP only, comprising discussion and documentation of resuscitate orders, advance directives, and patient questions about assisted suicide. These topics were approached and documented in all other consultations as well, however, not with the same level of detail. Advance directives were fixated or adapted at the patients' wish in 21% of all the consultations. A total of 9% of the patients reported to have considered the topic of assisted suicide, and 2% of patients had already fixed an appointment for an assisted suicide with the corresponding societies before the visit.

#### Network

An important subject of most consultations was the fortification of the patients' network. Table 5 shows which services were mediated in consultations. Frequently, the specialized palliative home care team was involved as a result of the palliative care consultation (*n* = 142, 39.1%). Social services were often included in the treatment plan as well. In addition, the interprofessional team informed the patients about further therapies such as physiotherapy, ergotherapy, nutritional therapy, spiritual guidance, psycho-oncology, music therapy, speech therapy, and complementary therapy.

**Table 5.** Newly Organized Additional Therapies and Network

	***n***	%
New network setup by the palliative care team after consultation		
Specialized palliative home care service	142	39.1
Family practitioner	12	3.3
Volunteers (nonmedical)	6	1.7
Relatives	1	0.3
Home care service	20	5.5
Hospice	12	3.3
Other institution	21	3.7
Social service	35	9.6

	***n***	%
New additional therapy after consultation		
Physiotherapy	8	2.2
Occupational therapy	5	1.4
Nutritional therapy	12	3.3
Spiritual guidance	3	0.8
Psycho-oncology	26	7.2
Music therapy	0	0.0
Logotherapy	3	0.8
Complementary medicine	10	2.8
others	4	1.1

Out of 556 consultations (*n* = 556; 100%; *ACP-only consultations* excluded).

ACP, advance care planning.

## Discussion

Early integration of palliative care is a recent emerging field that has proven to benefit patient outcomes and lower medical costs.^5,7,14^ However, the approaches to integrate early palliative care concepts simultaneously to standard care are not well defined yet, and due to different setups, published studies are often not comparable or small in sample size.^18^ In recent years, there has been growing interest in the use of outpatient palliative care clinics for patients in need of supportive care, observing an increase in referrals to this early-access services.^19^

In this context, the term of supportive instead of palliative care led to even more referrals, lowering the threshold for early referral.^20^ Indisputably, the value of referral to an outpatient clinic for end-of-life care of palliative patients has been confirmed, and consecutively, referral criteria for outpatient specialty palliative care have been defined in an international consensus article as additional assistance tool.^21–23^

Therefore, we aimed at addressing the benefits and areas of development of a palliative care outpatient clinic within a large university hospital in Switzerland. To our knowledge, this is one of the first studies describing a Swiss palliative care outpatient clinic, as a possible approach to early integration, implementing palliative support structures in an accessible and low threshold setting that also allows continuous follow-up and guidance in the disease trajectory. We characterize the patient population counseled for a period of five years and report on the main burdens and needs addressed in the consultations, as well as the large group of patients who did not present to their appointments. We identify a relevant symptom burden and distress in the presenting patients, and an overall late referral to our clinic.

The description of our patient population showed a predominantly oncological patient cohort, comprising ∼90% of cancer patients, despite a close cooperation with nononcological departments as cardiology or neurology, in the inpatient setting at our University Hospital. This is in accordance with other studies demonstrating a still existing gap regarding care of nononcological patients and confirms that palliative care offers do not reach all groups of patients equally depending on the setting.^24–26^ An approach to facilitate referral to palliative care for other departments is the use of checklists or identification of triggers.^11,27–29^

However, definition of specific triggers or characteristics that identify patients qualifying for a referral to palliative care is complicated by the heterogeneity of patients and the variety of needs.^29^ In addition, the referring physicians must identify said needs within their consultations first, even if unmentioned by the patient. Often, this occurs when needs and burdens become apparent by worsening, when several problems cumulate, or when visits become apparently frequent as a surrogate marker for an unstable situation at home.

Accordingly, we show that more than one symptom in the ESAS was reported in 73.7% of consultations, and 162 (29.1%) of patients required external support from home care services due to insufficient self-care capacities (Fig. 2, Tables 3, 5, and Supplementary Table S2). Pain was the most predominantly reported single symptom in the entire patient population independent of sex (53.5% for men and 56.7% for women, respectively; *p* = 0.120), yet less prevalent in the group of noncancer patients compared with cancer patients (56.0% vs. 39.1%, respectively; *p* = 0.037) (Table 1 and Supplementary Table S2).

Symptom burden differentiated based on the primary leading symptom (Table 2). Clustering of symptoms revealed that gastrointestinal symptoms were also high-ranked burdening symptoms (Fig. 2). On psychosocial aspects, patients and caregivers benefitted from assistance in implementation of home care services and home care palliative care nursing services; especially support of specialized palliative home care was organized frequently (39.1%) (Table 5). On the one hand, women in our population were significantly more often living alone, therefore benefitting from support directly; on the other hand, most caregivers to men or women were female, and also in need for support in their caregiver activity (Supplementary Table S2).

Overall, the main addressed topics during the consultations did not differ from those of other palliative care outpatient clinics.^30^ Solely spiritual distress was hardly ever addressed or documented by patients or the interprofessional team, in contrast to other publications.^31^

With regard to timing, we observed an overall late referral in our outpatient population of supposedly early palliative patients in ambulatory setting (Table 4). In The overall cohort, referral to our consultation still occurred as late as within four months in median before death. This is even more intriguing considering that this late referral was independent of the length of disease trajectory and of the primary diagnosis.

Considering the recommendations from the American Society of Clinical Oncology, which recommends that all cancer patients should be offered palliative care within eight weeks from diagnosis of advanced disease, and that ACP should be discussed with all patients with a life expectancy less than a year, our outpatient population was referred clearly at a very late stage, and presented already with substantial distress and symptom load (Tables 2 and 3).^32^

This delaying of referral in the context of the attempt to early integration of palliative care currently represents a major challenge, especially when palliative care is still stigmatized as a pure end-of-life care reserved to patients with no remaining disease-specific therapy options.^1^ It also limits the options to prepare patients und provide support in an adequate manner, despite well-described beneficial results of early outpatient referral.^21^

Admittedly, identifying the right moment for referral in the trajectory of disease remains difficult despite guidelines. The uncertainty of prognostication in the literature underlines this fact. In this light, Kamal et al. propagates a prognosis-independent referral culture, which might facilitate the process.^2^ In view of the overall late referral in our cohort of oncological and nononcological patients with heterogeneous prognosis, this concept would alleviate the decision-making process for the referring physicians.

Zimmermann et al. recommend referring a patient with advanced cancer disease when they have an ECOG Performance Status of at least 0 to 2 and a prognosis of 6 to 24 months, mainly based on their experience that referrals for palliative care outpatient consultation usually occurred only in the past two months before death, which is consistently considered too late for patients and caregivers to benefit.^33^ In our overall patient population, median survival from referral to death was four months, and, therefore, also late to benefit from supportive offers. In this context, we also investigated the relatively high rate of cancellations and no-shows in our outpatient service (Supplementary Table S1).

We identified a cancellation and no-show rate of 292 consultations (31.3%) for 244 patients, of which most no-shows remained for unknown reasons (*n* = 115, 39.4%). However, emergency hospitalizations occurring before the outpatient clinic appointment could take place was the second most common reason for no-show in 82 patients (28.1%), and death occurring before the outpatient clinic appointment could take place was the reason for no-show in 39 patients (13.4%). These patients were clearly referred to our clinic too late.

This study has some limitations. First, due to the retrospective nature of the study, some variables such as performance status were often not consistently documented limiting the available data for an evaluation and analysis. In addition, with patient-centered consultations, standardization of the consultation approach is only possible to a certain extent, as by use of the SENS Model for conversational setup^17^; still, needs and burden differ between patients, and, therefore, consultations are not strictly comparable.

Likewise, although patient-reported outcomes were collected on a regular level by means of ESAS, no longitudinal quality-of-life assessments were collected to validate a benefit of the consultations for patients or caregivers, and especially the needs of the later might have been under-reported in the documentation. Finally, although the larger part of the outpatient population was indeed oncological, the nononcological patients were assessed by means of ESAS and distress thermometer as well, despite these tools being validated for oncological populations primarily.

However, we have demonstrated a successful implementation and feasibility of a palliative care outpatient clinic integrated in the body of a university hospital, in close cooperation with referring departments. We present >600 consultations over a span of five years, in which patient-reported outcomes were collected. The structure of our clinic combining different and broad services (inpatient palliative care ward, inpatient palliative care consult service for other departments, and outpatient clinic) enables a palliative care offer throughout the trajectory of the disease from early stages of the disease to end-of-life care, which allows for a solid continuity of care from the interprofessional palliative care team.^34^

## Conclusions

The implementation of an outpatient palliative care clinic for early integration of supportive care simultaneously to disease-specific treatments adds to the increasing and developing field of patient-centered care. Optimizing home care early and easing the transition to inpatient palliative care later on allows for a holistic and continuous care for oncological and nononcological palliative patients. Patient, caregiver, physician, and the health care system can benefit of this form of early palliative care support, independent of the origin of the disease. The use of palliative-targeted patient-reported outcome measures, as the integrated palliative care Outcome Scale might be an improvement to consider a better inclusion of palliative patients of noncancer origin as well, which are currently under-represented in our service.

## Supplementary Material

Supplemental data

Supplemental data

Supplemental data

## Data Availability

Collected patient data are confidential and not available for publication.

## Abbreviations Used

ACP: advance care planning
ESAS: Edmonton Symptom Assessment System
NCCN: National Comprehensive Cancer Network
USZ: University Hospital of Zurich
