# Supplementary material for: Outpatient Palliative Care Service Involvement: A Five-Year Experience from a Tertiary Hospital in Switzerland
Source: Palliat Med Rep. 2024 Jan 5;5(1):10–9. doi: 10.1089/pmr.2023.0052 (PMC10797309; doi:10.1089/pmr.2023.0052)
Supplement: Supplemental data [file Suppl_TableS1.docx]

**Supplementary Table 1. Patient characteristics (show versus no-show)**

|  | Visit  (N=363)  N (%) | No-show  (N=244)  N (%) | p-value |
| --- | --- | --- | --- |
| Age (years)  Median  Range | 65  19-94 | 62.7  18-94 | 0.980 |
| Age groups  <50  50-69  70-79  >80 | 53 (14.6)  110 (30.3)  92 (25.3)  108 (29.8) | 37 (15.4)  110 (45.6)  59 (24.5)  35 (14.5) | **<0.001** |
| Sex  Male  Female | 208 (57.3)  155 (42.7) | 140 (57.4)  104 (42.6) | 0.985 |
| Class of Insurance  General  Semi-private  Private  Missing | 212 (58.6)  2 (0.6)  148 (40.9)  1 | 125 (53.9)  1 (0.4)  106 (45.7)  12 | 0.509 |
| Nationality  Swiss  German  Italian  Other  Missing | 288 (79.3)  25 (6.9)  15 (4.1)  35 (9.6)  0 | 187 (80.3)  11 (4.7)  9 (3.9)  26 (11.2)  11 | 0.691 |
| Confession  Catholic  Protestant  Muslim  Jewish  Other  None  Missing | 111 (32.3)  126 (36.6)  8 (2.3)  1 (0.3)  32 (9.3)  66 (19.2)  19 | 65 (29.3)  75 (33.8)  10 (4.5)  1 (0.5)  18 (8.1)  53 (23.9)  22 | 0.489 |
| Main diagnosis  Oncology  Brain cancer  Head and Neck cancer  Lung cancer  Gyneco-Oncology  Gastro-intestinal cancer  Prostate cancer  Sarcoma  Dermato-Oncology  Nephro-Uro-Oncology  Hemato-Oncology  Other cancer*  Non-Oncology  Cardiology  Pneumology  Neurology  Other non-cancer**  Missing | 50 (13.8)  42 (11.6)  64 (17.6)  28 (7.7)  50 (13.8)  22 (6.1)  17 (4.7)  21 (5.8)  18 (5.0)  19 (5.2)  10 (2.8)  5 (1.4)  4 (1.1)  6 (1.7)  7 (1.9)  0 | 34 (14.7)  25 (10.8)  27 (11.7)  26 (11.3)  35 (15.2)  15 (6.5)  13 (5.6)  16 (6.9)  18 (7.8)  7 (3.0)  5 (2.2)  1 (0.4)  1 (0.4)  4 (1.7)  4 (1.7)  13 | 0.622 |
| Diagnosis  Oncological  Non-oncological | 341 (93.9)  22 (6.1) | 221 (92.9)  10 (7.1) | 0.360 |

*other cancer including Cancer of unknown primary (CUP), neurofibromatosis, germ cell cancer, chondroma

**other non-cancer including anorexia, chronic pain syndromes, systemic lupus erythematodes, thymoma, polyangiitis, cirrhosis, myelofibrosis and severe diabetes
