# Supplementary material for: Outpatient Palliative Care Service Involvement: A Five-Year Experience from a Tertiary Hospital in Switzerland
Source: Palliat Med Rep. 2024 Jan 5;5(1):10–9. doi: 10.1089/pmr.2023.0052 (PMC10797309; doi:10.1089/pmr.2023.0052)
Supplement: Supplemental data [file Suppl_TableS2.docx]

**Supplementary Table 2. Group comparison by sex (consultation cohort)**

|  | Male  (N=208)  N (%) | Female  (N=155)  N (%) | p-value |
| --- | --- | --- | --- |
| Age (years)  Median  Range | 66.5  24.0 – 98.0 | 66.0  19.0-94.0 | 0.057 |
| Age groups  <50  50-69  70-79  >80 | 31 (14.9)  60 (28.8)  52 (25.0)  65 (31.3) | 22 (14.2)  50 (32.3)  40 (25.8)  43 (27.7) | 0.859 |
| Diagnosis  Oncological  Non-oncological | 196 (94.2)  12 (5.8) | 144 (92.9)  11 (7.1) | 0.666 |
| Main diagnosis  Oncology  Brain cancer  Head and Neck cancer  Lung cancer  Gyneco-Oncology  Gastro-intestinal cancer  Prostate cancer  Sarcoma  Dermato-Oncology  Nephro-Uro-Oncology  Hemato-Oncology  Other cancer*  Non-Oncology  Cardiology  Pneumonoly  Neurology  Other non-cancer** | 34 (16.3)  27 (13.0)  40 (19.2)  0 (0)  26 (12.5)  22 (10.6)  10 (4.8)  8 (3.8)  13 (6.3)  12 (5.8)  5 (2.4)  3 (1.4)  4 (1.9)  3 (1.4)  1 (0.5) | 16 (10.3)  15 (9.7)  24 (15.5)  28 (18.1)  24 (15.5)  0 (0)  7 (4.5)  13 (8.4)  5 (3.2)  7 (4.5)  5 (3.2)  2 (1.3)  0 (0)  3 (1.9)  6 (3.9) | **<0.001** |
| Main symptom  Pain  Dyspnea  Fatigue  Neurological  Psycho-emotional  None  Other | 107 (53.5)  18 (9.0)  29 (14.5)  23 (11.5)  15 (7.5)  8 (4.0)  8 (-) | 85 (56.7)  7 (4.7)  17 (11.3)  18 (12.0)  21 (14.0)  2 (1.3)  5 (-) | 0.120 |
| Symptom load  Little  Moderate  Strong  Extreme  Missing | 31 (15.5)  109 (54.5)  54 (27.0)  6 (3.0)  8 (-) | 29 (20.3)  70 (49.0)  40 (28.0)  4 (2.8)  12 (-) | 0.655 |
| Living situation  Alone  Supported | 48 (23.1)  160 (76.9) | 56 (36.1)  99 (63.9) | **0.007** |
| Advance directives  Pre-existent  Within consultation  None  Missing | 100 (55.2)  32 (17.7)  49 (27.1)  27 (-) | 69 (52.7)  22 (16.8)  40 (30.5)  24 (-) | 0.800 |

*other including anorexia, chronic pain syndromes, systemic lupus erythematodes, polyangiitis, cirrhosis, thymoma, myelofibrosis and severe diabetes
