# Supplementary material for: Outpatient Palliative Care Service Involvement: A Five-Year Experience from a Tertiary Hospital in Switzerland
Source: Palliat Med Rep. 2024 Jan 5;5(1):10–9. doi: 10.1089/pmr.2023.0052 (PMC10797309; doi:10.1089/pmr.2023.0052)
Supplement: Supplemental data [file Suppl_TableS3.docx]

**Supplementary Table 3. Survival data from diagnosis (log rank analysis)**

|  | |  | | | |
| --- | --- | --- | --- | --- | --- |
| **Survival** |  | N (events) | Median OS (months) | 95% CI | p-value |
| **All patients** |  | 361 (259) | 34.0 | 28.12-39.81 |  |
| **Age group** | < 50 | 52 (36) | 51.0 | 41.24-60.76 | **0.037** |
|  | 50-59 | 110 (82) | 29.0 | 18.70-39.21 |  |
|  | 60-69 | 91 (75) | 27.0 | 15.64-38.37 |  |
|  | $\geq$ 70 | 108 (66) | 36.0 | 27.15-44.86 |  |
| **Sex** | male | 206 (152) | 32.0 | 23.97-40.06 | 0.071 |
|  | female | 155 (107) | 36.0 | 24.87-47.13 |  |
| **Diagnosis** | Non-oncological | 23 (15) | 129.0 | 15.81-242.19 | **<0.001** |
|  | Oncological | 338 (244) | 32.0 | 25.57-38.43 |  |
| **Main symptom** | pain | 190 (140) | 38.0 | 29.33-46.68 | 0.998 |
|  | dyspnea | 25 (18) | 32.0 | 6.06-57.94 |  |
|  | fatigue | 46 (32) | 27.0 | 12.52-41.49 |  |
|  | Neurological | 41 (31) | 30.0 | 18.60-41.41 |  |
|  | Psycho-emotional | 36 (24) | 30.0 | 14.64-45.36 |  |
|  | None | 10 (7) | 23.0 | 4.41-41.59 |  |
| **Symptom load** | Little | 60 (42) | 36.0 | 26.90-45.01 | 0.904 |
|  | Moderate | 178 (128) | 31.0 | 34.09-38.91 |  |
|  | Strong | 94 (73) | 30.0 | 17.76-42.24 |  |
|  | Extreme | 9 (7) | 37.0 | 16.33-57.67 |  |
| **Living situation** | Alone | 104 (73) | 38.0 | 25.41-50.59 | 0.387 |
|  | Supported | 257 (186) | 32.0 | 24.37-39.64 |  |
| **Advance directives** | Pre-existent | 168 (126) | 35.0 | 25.59-44.41 | 0.304 |
|  | Within consultation | 53 (36) | 34.0 | 23.01-44.92 |  |
|  | None | 89 (64) | 30.0 | 20.18-39.82 |  |
